# Authors’ Reply: Concerns on Generalizability

**DOI:** 10.2196/51852

**Published:** 2023-09-21

**Authors:** Hsueh-Wen Chung, Chen-Jei Tai, Polun Chang, Wen-Lin Su, Li-Yin Chien

**Affiliations:** 1 Department of Nursing College of Nursing National Yang Ming Chiao Tung University Taipei City Taiwan; 2 Department of Obstetrics and Gynecology School of Medicine, College of Medicine Taipei Medical University Taipei City Taiwan; 3 Tai's Traditional Chinese Medicine Clinic Taipei City Taiwan; 4 Institute of Biomedical Informatics National Yang Ming Chiao Tung University Taipei City Taiwan; 5 Division of Pulmonary and Critical Care Medicine, Department of Internal Medicine Taipei Tzu Chi Hospital Buddhist Tzu Chi Medical Foundation New Taipei City Taiwan; 6 School of Medicine Tzu Chi University Hualien Taiwan; 7 Institute of Community Health Care College of Nursing National Yang Ming Chiao Tung University Taipei City Taiwan

**Keywords:** mHealth app, mobile health, mHealth, app, prediabetes, traditional Chinese medicine, TCM, health-related quality of life, body constitution, meridian energy

We appreciate the thoughtful comments by Lin [1] on our study [2]. We have mentioned that the limitations of our study include a small sample size and a short follow-up period. We also suggested that future studies should be conducted with a larger sample size and a longer follow-up period.

The first comment raised was regarding the use of a randomized controlled trial (RCT) design without incorporating propensity score (PS) matching. Kuss et al [3] indicated that PS cannot take into account factors that are unknown or were not measured and, therefore, is more suitable for observational studies. An RCT is the only design that can ensure equal distributions of unknown confounding factors, and it enables the making of causal statements on treatment effects. Although in recent years a few studies have suggested the use of PS in RCTs [4], we are still uncertain about its appropriateness for RCTs. Future studies may attempt to investigate its use in RCTs. Nonetheless, following your suggestion, we tried regression adjustment with PS. The results appeared to be similar to those we presented in the paper.

The second comment suggests that we provide descriptive data for both between- and within-group comparisons. In addition, in cases where population characteristics are unknown, the Wilcoxon rank-sum test could have been considered for analysis. In our paper, we presented data for between- and within-group comparisons in Multimedia Appendix 5. We checked the distribution of the outcome variables including yang-deficiency, yin-deficiency, phlegm-stasis body constitution, body energy, and physical and mental component scores; the distribution approximated a normal distribution in our study. According to the central limit theorem, when the sample size of each group is greater than 30, it is reasonable to assume that the distribution of the sample means approaches normality [5].

The third point raised pertains to the absence of a prespecified subgroup analysis in our study. Owing to the predetermined objectives, hypothesis, and statistical analysis methodology at the initial stages of the study, coupled with the limitation of a small sample size, we did not incorporate a prespecified subgroup analysis. It is suggested that future studies, with an increase in sample size, may consider carrying out such an analysis.

Once again, we appreciate this opportunity to clarify our study. Such dialogues enable the identification and discussion of more aspects of this important issue.

## Appendix

Multimedia Appendix 1 Comparison of the primary and secondary outcomes among the TCM mHealth app, ordinary mHealth app, and control groups (N=121).

## Abbreviations

PS: propensity score
RCT: randomized controlled trial
